# Therapeutic Potential of Butyrate for Treatment of Type 2 Diabetes

**DOI:** 10.3389/fendo.2021.761834

**Published:** 2021-10-19

**Authors:** Tulika Arora, Valentina Tremaroli

**Affiliations:** ^1^ Novo Nordisk Foundation Center for Basic Metabolic Research, Faculty of Health and Medical Sciences, University of Copenhagen, Copenhagen, Denmark; ^2^ The Wallenberg Laboratory, Department of Molecular and Clinical Medicine, Institute of Medicine, Sahlgrenska Academy, University of Gothenburg, Gothenburg, Sweden

**Keywords:** type 2 diabetes (T2D), microbiota, butyrate, metabolic disease, short chain fatty acids (SCFAs)

## Abstract

Metagenomics studies have shown that type 2 diabetes (T2D) is associated with an altered gut microbiota. Whereas different microbiota patterns have been observed in independent human cohorts, reduction of butyrate-producing bacteria has consistently been found in individuals with T2D, as well as in those with prediabetes. Butyrate is produced in the large intestine by microbial fermentations, particularly of dietary fiber, and serves as primary fuel for colonocytes. It also acts as histone deacetylase inhibitor and ligand to G-protein coupled receptors, affecting cellular signaling in target cells, such as enteroendocrine cells. Therefore, butyrate has become an attractive drug target for T2D, and treatment strategies have been devised to increase its intestinal levels, for example by supplementation of butyrate-producing bacteria and dietary fiber, or through fecal microbiota transplant (FMT). In this review, we provide an overview of current literature indicating that these strategies have yielded encouraging results and short-term benefits in humans, but long-term improvements of glycemic control have not been reported so far. Further studies are required to find effective approaches to restore butyrate-producing bacteria and butyrate levels in the human gut, and to investigate their impact on glucose regulation in T2D.

## Introduction

Type 2 diabetes (T2D) is a global concern and is projected to affect 700 million people by 2045 (1). Although lifestyle interventions (including diet, exercise and weight loss) are the first option for managing T2D, patients are often prescribed medications. A variety of drugs are already available, but side effects (such as pancreatitis and gastrointestinal complications) (2), and lifelong dependency on drugs entail a significant burden on the patients and on the healthcare system globally. Therefore, novel individualized therapies are being developed, focused on safety and personalized management of T2D.

‘Gut microbiota’ is a term used to describe the trillions of microbes that live in the gastrointestinal tract. The gut microbiota has been identified as a virtual organ interacting locally with the gut and systemically with other organs in the host to facilitate multiple physiological processes (3). The interest in understanding the composition and function of the gut microbiota has increased exponentially over the last two decades. From the initial studies addressing the possibility to culture and profile gut microbiota communities (4–7), the field has developed to describe the core human gut microbiota (8–12), its tremendous genetic potential (100 times larger than the human genome) (10, 13) and variations according to geographical location (14, 15), ethnicity (16, 17) and disease status (18), including T2D (19, 20). How an altered gut microbiota can impact metabolic health is debated, but metabolomics investigations have shown that the gut microbiota contribute to the variation of blood metabolites in humans (21), with important implications for metabolic regulation (22). Trimethylamine oxide (23), imidazole propionate (24) and indolepropionic acid (25) are examples of microbial metabolites associated with development or protection against metabolic diseases, and their specific roles in metabolic diseases as well as signaling mechanisms are currently under investigation.

**Figure 1:**
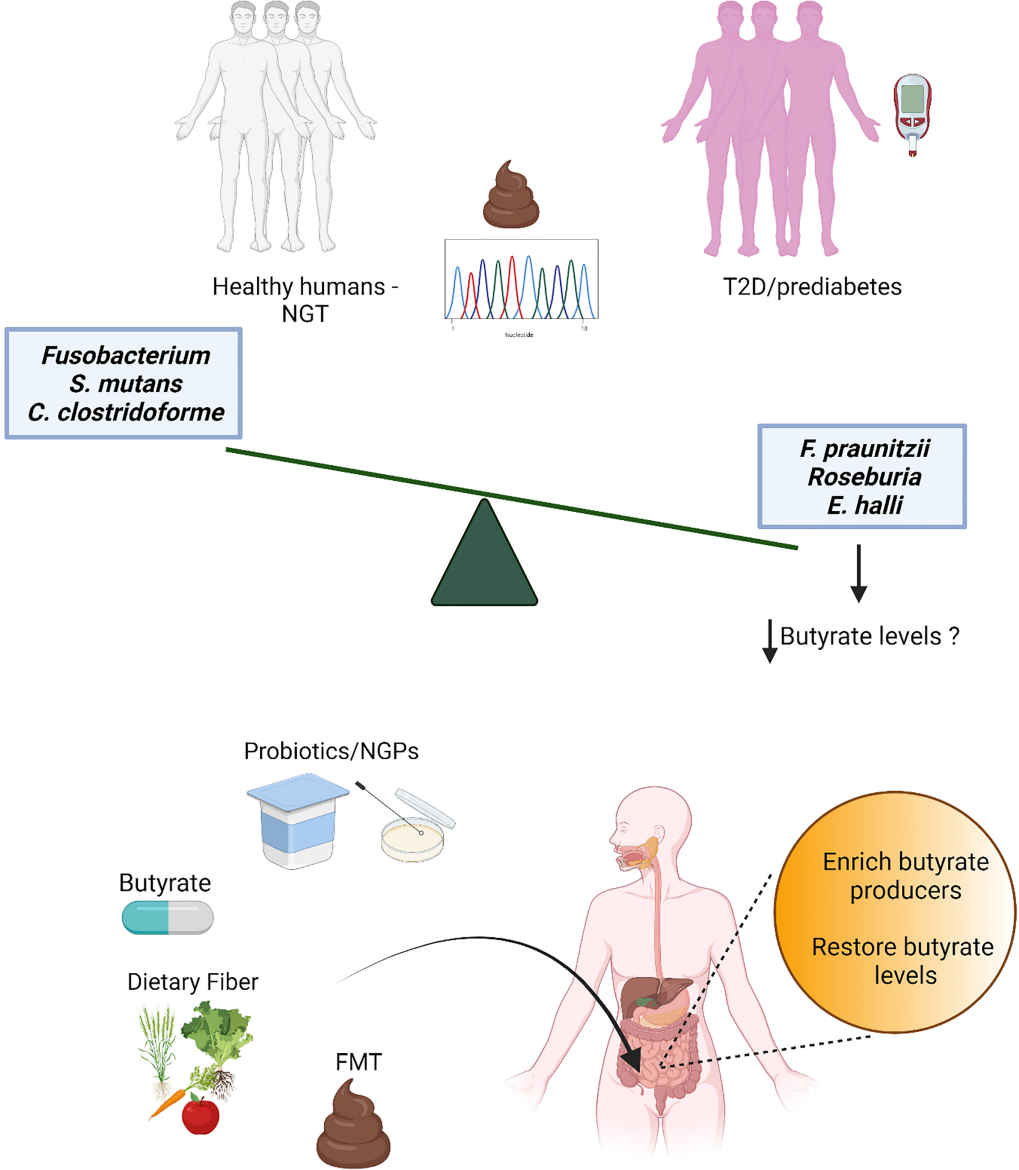
Several independent metagenomics studies have detected a decrease of butyrate producers in stools from individuals with prediabetes and type 2 diabetes (T2D) compared to individuals with normal glucose tolerance (NGT). While the loss of butyrate producers is robust and associated also with obesity and other cardiometabolic comorbidities (18), consistent patterns for increased microbial features have not been found, possibly due to the redundancy of the gut microbiota and stochasticity in gut microbiota alterations (30); only few gut microbiota species (mostly opportunistic pathogens) have been observed as increased in a limited number of studies. Thus, butyrate and butyrate producers have been selected as potential targets for the development of novel therapeutic strategies for T2D, such as direct butyrate administration, administration of butyrate producers and/or bacteria able to promote intestinal butyrate production [probiotics and next-generation probiotics (NGPs)], interventions with dietary fibers and fecal microbiota transplant (FMT).

Butyrate is one of the short chain fatty acids (SCFAs) produced as end-products of intestinal microbial fermentations (26, 27). Butyrate is absorbed rapidly in the gut and acts as signaling molecule in receptor-mediated signaling in numerous cell types (28). Microbial butyrate production in the human gut has been known for decades (29) before the large sequencing efforts of the gut microbiota started, but it was only in the last decade that metagenomics surveys consistently revealed in multiple independent human cohorts a decrease of butyrate-producing bacteria in individuals with T2D (19, 20). As restoration of butyrate-producing bacteria and butyrate levels might provide new treatment options for T2D, here we review recent literature on the association of butyrate and butyrate-producing bacteria with T2D, and discuss the therapeutic potential for management and treatment of this disease (**Figure 1**).

## Type 2 Diabetes and Butyrate-Producing Bacteria

T2D has been associated with compositional and functional shifts in the gut microbiota. One of the striking features that was consistently observed in multiple cohorts across diverse geographical locations is the reduction of butyrate-producing bacteria in individuals with T2D. The first observations came from shotgun metagenomics studies of fecal communities in Chinese (20) and Swedish (19) individuals, and showed decreased abundance of several butyrate producers such as *Roseburia* and *Faecalibacterium prausnitzii*; these findings were later extended to individuals with T2D in Indian (31) and African (32) populations. Lower abundance of *F. prausnitizii* has also been found in the mesenchymal adipose tissue of obese individuals with T2D compared to normoglycemic controls matched for body mass index (33), reflecting the lower intestinal abundance of this bacterium in T2D independent of obesity, but linked to the metabolic status. The positive association between microbial potential for butyrate production and normoglycemia is supported by the results of a Dutch study that combined fecal microbiota metagenomics and human genome sequencing, and showed that higher potential for butyrate production driven by host genetics was linked to improved insulin response to an oral glucose load in normoglycemic individuals (34). Furthermore, one recent study has shown that also the diurnal oscillation of the gut microbiota is altered in T2D, and in particular that the abundance of several bacteria, among which *Roseburia* and *F. prausnitzii*, lost rhythmicity, and that the arrhythmicity might be important for risk classification and prediction of T2D (35). However, it is important to mention that other gut commensals (e.g. *Akkermansia, Bifidobacterium* and *Bacteroides*) are also decreased in T2D and might play additional roles for the modulation of gut barrier function, inflammation and metabolism, as reviewed elsewhere (36, 37).

The initial studies on the associations between gut microbiota and T2D did not account for medications used for T2D treatment; however, it is now established that numerous non-antibiotic drugs can influence the gut microbiota (38). Metformin is the first line of treatment in newly discovered T2D patients and was first demonstrated to influence gut microbiota composition in diet induced obese mouse models, where it resulted in enrichment of *Akkermansia muciniphila* (39, 40), a gut commensal showing beneficial effects on metabolism (41). Similar results were later observed also in large human cohorts (including both treatment naïve and metformin-treated T2D patients), in which shotgun sequencing of the fecal microbiota showed enrichment of butyrate producers, and in some cases also of *A. muciniphila* (42–45). By using germ-free (GF) mice, it was also shown that part of the antidiabetic effects of metformin can be explained by the microbiota shifts. GF mice are devoid of any microbiota, and are an efficient model to understand the functional significance of compositional changes in the gut microbiota: fecal microbiota transplant from metformin-treated individuals to GF mice were able to transfer the improved glucose tolerance phenotype to the recipients (45). In addition, gut microbiota has also been shown to interact with metformin and the diet to promote microbial synthesis of agmatine, specifically in individuals with T2D, an effector molecule able to regulate host lipid metabolism (46). Overall, these results indicate a possible beneficial role of the microbial shifts induced by metformin for the improvement of the glycemic and metabolic status in T2D. However, these studies also indicate that potential confounders [e.g., medications, dietary supplements and obesity (47)] should be considered in gut microbiota analyses for the identification of possible microbial signatures of T2D.

T2D is a chronic metabolic disorder that can remain undetected for a number of years. Prediabetes precedes T2D, and often presents with intermediate hyperglycemia, such as impaired fasting glucose (IFG), impaired glucose tolerance (IGT) or combined glucose intolerance (CGI) (48). Microbiota profiling in a Danish cohort diagnosed with prediabetes revealed lower abundance of butyrate-producing bacteria compared with age- and sex-matched individuals with normal glucose regulation (49). In addition, a metagenomic study in a T2D-treatment-naïve Swedish cohort has shown that microbiota composition is altered in IGT and CGI, and is characterized by a reduction in the abundance of butyrate-producing bacteria and the terminal genes for butyrate synthesis (43). These studies indicate that the butyrate-producing potential of the gut microbiome is depleted already in the prediabetes state, and suggest that replenishment of butyrate producers or butyrate levels might be important to delay or prevent progression to T2D. In contrast with the consistent finding of reduced abundance of butyrate-producing bacteria in T2D, different studies report extensive variation for the levels of butyrate in feces and/or blood of individuals with T2D compared to controls. In the large Dutch cohort, fecal butyrate levels did not correlate with either butyrate production potential or the selected anthropometric and glycemic traits, thus suggesting that fecal butyrate levels might not be representative of butyrate production and absorption (34). However, results from two smaller cohorts have showed decreased levels of SCFAs (including butyrate) in individuals with T2D as well as significant correlation with metabolic parameters (50, 51). Therefore, as butyrate is volatile and quickly absorbed and consumed by the colonic epithelium, static measurements in fecal and blood samples might not be sufficient to reveal an actual reduction of butyrate levels in T2D. Radioactive tracers might offer a better solution to trace the origin and absorption of butyrate, but would not be applicable to large-scale human cohort studies.

## Butyrate – Production, Absorption and Physiological Roles

The SCFAs acetate, propionate and butyrate are produced by microbial fermentations in the gut (52). Most of the butyrate producers are distributed within multiple clusters of Clostridia, in the phylum Firmicutes (53). Butyrate is primarily a product of carbohydrate fermentation produced by condensation of two acetyl CoA molecules. However, lactate and by-products of amino acids metabolism are also cross-fed to generate butyrate (54). Two key enzymes have been identified for butyrate production by the gut microbiota from carbohydrates: the butyryl CoA:acetate CoA transferase (*but*) is the primary enzyme, whereas the butyrate kinase (*buk*) is present in a few strains (55). The terminal enzymes for butyrate production from amino acids are also known [i.e., butyryl CoA:4-hydroxybutyrate CoA transferase (*4-hbt*) and butyryl CoA:acetoacetate CoA transferase (*ato*)] (56), but their abundance in the human gut is lower compared to *but* and *buk*.

In humans, the major site of SCFAs and butyrate production is the colon, from where total SCFAs are drained into the portal blood with much higher concentration (375µmol/l) than in peripheral blood (79µmol/l) (29). However, approximately 95% of the butyrate produced in the gut lumen is rapidly absorbed by colonocytes and fuels cellular metabolism through mitochondrial β-oxidation (57). Colonic delivery of ^13^C labelled acetate, propionate and butyrate in healthy subjects revealed that only 36% of the acetate, 9% of the propionate and 2% of the butyrate could be recovered in blood samples collected over different time points during the day (58). Interestingly, 24% of acetate was bio-converted to butyrate, but the authors did not find significant correlation between the percentage of interconversion and the gene copies of *but* or *buk* in the fecal samples (58). These results confirm the observations from the large Dutch cohort (34), and thus indicate that the abundance of the genes does not fully reflect the activity, as different intestinal variables (e.g., pH, redox potential, lactate and acetate levels) can influence the rates of butyrate production (52).

In the GF mouse model, colonocytes are in a state of nutrient deficiency that causes cellular autophagy in the colonic epithelium (59) and slower intestinal transit (60). Supplementation of butyrate reverses these phenotypes (i.e. prevents autophagy and decreases intestinal transit time), indicating that microbially-produced butyrate is an important source of energy for colonic epithelial cells and its deficiency results in reversible adaptive mechanisms to cope with nutrient deficiency. In addition, mouse studies have shown that butyrate contributes to maintain the colonic environment anaerobic through the activation of the peroxisome proliferator-activated receptor-gamma in colonocytes, which results in the induction of β-oxidation (57). This process has been shown to consume oxygen and prevent colonic invasion by pathogenic *Salmonella* and *Escherichia* species (57). Because of its effects on colonocytes, butyrate levels in the gut might be important not only for signaling to the host (as described below) but also for the composition of the gut microbiota. Therefore, butyrate might be a particularly interesting therapeutic target.

Butyrate also serves as histone deacetylase (HDAC) inhibitor to regulate the expression of genes by epigenetic mechanisms. Supernatants from cultures of butyrate-producing bacteria from the human gut microbiota express HDAC inhibitory activity to class I and II HDACs (61). T2D is associated with epigenetic changes in multiple organs (62) and it seems plausible that microbially-produced butyrate could be one of the contributing factors. In a non-obese diabetic mouse model, it has been observed that the reduction of intestinal butyrate associated with T2D caused an increase in colonic HDAC activity resulting in production of reactive oxygen species and alteration of colonic permeability (63). Furthermore, supplementation of sodium butyrate has been shown to ameliorate palmitate-induced insulin resistance in muscle cells by promoting hyperacetylation of insulin receptor substrate-1 in an *in vitro* study (64). Consistent with this study, supplementation of sodium butyrate to mice modulated mitochondrial chromatin structure (65) and lowered HDAC activity in skeletal muscle resulting in improved insulin sensitivity (66). Finally, sodium butyrate treatment has been shown to suppress HDAC activity also in mouse liver, leading to reduced gluconeogenesis and improved glucose homeostasis (67). These animal studies suggest that reduction of butyrate in T2D may alter gene expression by epigenetic mechanisms leading to insulin resistance, which can be reversed by butyrate supplementation. However, this observation awaits validation in human cohorts.

Butyrate (as well as the other SCFAs) also acts as a signaling molecule and is identified as a ligand for G-protein coupled receptors (GPCRs), such as FFAR3, FFAR2 and GPR109A. Human orthologs of FFAR2 have similar affinity for acetate and propionate followed by butyrate, while FFAR3 has higher affinity for propionate and butyrate compared to acetate (68). Niacin is identified as the most potent ligand of GPR109A, though butyrate also shows weak binding (69). These receptors are located in distal regions of the intestine and in adipocytes (70). In the intestinal lumen, the concentrations of SCFAs are supramaximal and therefore it is thought that these receptors are localized on the basolateral side of the intestinal epithelium (70). FFAR3 and FFAR2 are present on hormone-producing enteroendocrine cells (EECs): exposure to SCFAs stimulates EECs differentiation (71), and binding of SCFAs to FFAR3 and FFAR2 results in altered gene expression and secretion of gut hormones, such as peptide YY (PYY) and glucagon like peptide-1 (GLP-1) (72, 73). GLP-1 is one of the gut hormones that profoundly affects glucose regulation by promoting post-prandial insulin secretion, and GLP-1 based drugs are approved for the treatment of T2D (74). Supplementation of butyrate along with inulin have been shown to increase GLP-1 levels in individuals with T2D with significant improvement in the glycemic status (75). However, it is important to note that the *in vitro* studies mentioned here report different effects after stimulation with a mix of SCFAs or with individual SCFAs, indicating that the effects on EECs are not exclusively mediated by butyrate. Nevertheless, in human cell lines, butyrate selectively stimulates PYY secretion through mechanisms largely driven by HDAC inhibition (76).

In addition to signaling in the gut, activation of SCFAs-binding receptors might be important also in the adipose tissue. Selective chemical agonism of GPR109A in individuals with T2D decreased fasting glucose, but not Hb1Ac, through inhibition of lipolysis in adipocytes as demonstrated by the decreased circulating levels of non-esterified fatty acids in the patients (77, 78). Consistent with these results, overexpression of FFAR2 in adipose tissue protected mice from gut microbiota-dependent diet-induced obesity (79). Additionally, a role for butyrate in the stimulation of thermogenesis in brown and white adipose tissue has been demonstrated in rodents (66), with potential relevance for the regulation of glycaemia. However, oral supplementation of butyrate in individuals with T2D did not alter brown adipose tissue activity (80).

Finally, butyrate signaling might also be important for islets function. *In vitro* experiments have showed that addition of butyrate in culture media reduced streptozotocin-induced islet cell death (81). Moreover, supplementing HFD with 5% butyrate in a T2D mouse model prevented β-cell expansion and fat accumulation in the pancreas (82). In contrast, an oral dose of 4g butyrate to individuals with type 1 diabetes for a month did not improve β-cell function or islet autoimmunity (83).

**Figure 2:**
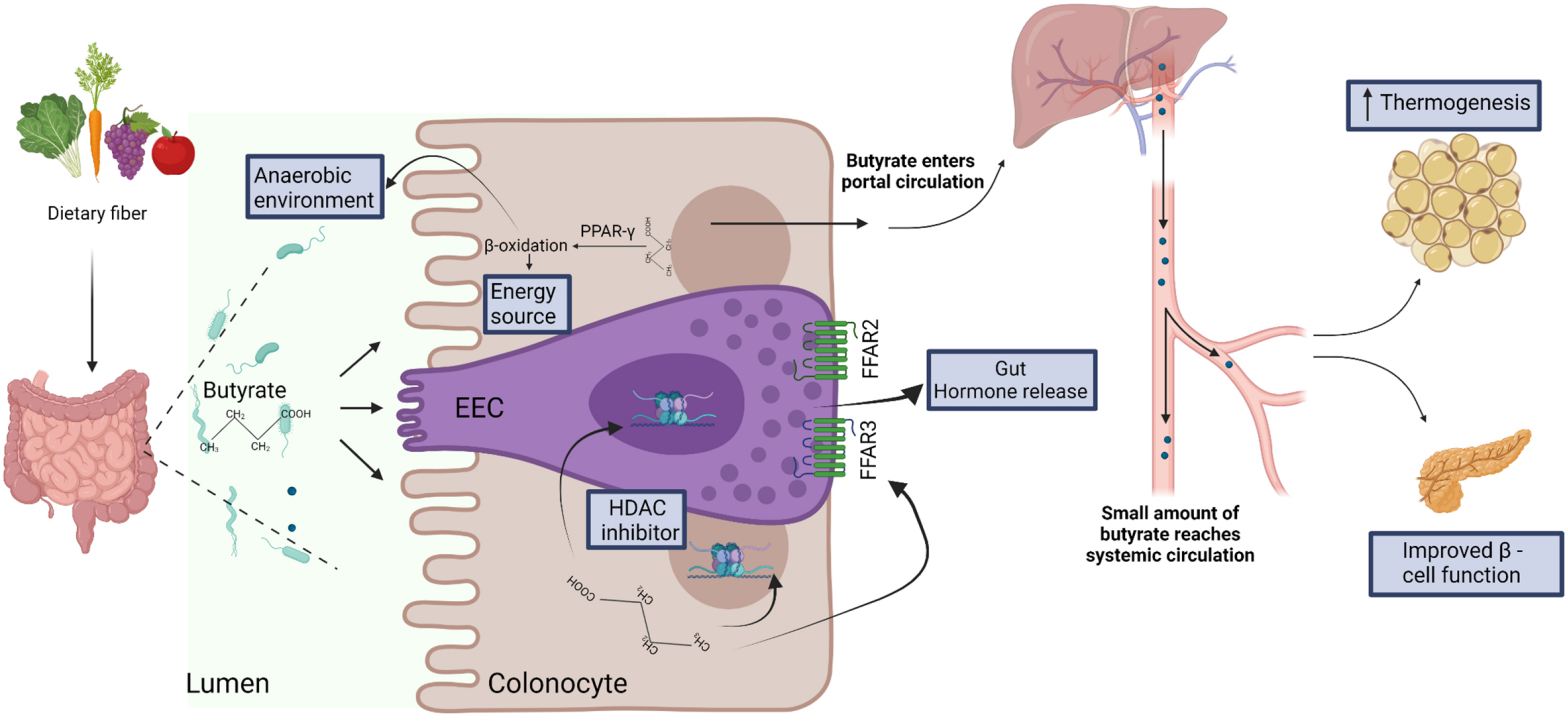
Dietary fiber is fermented by the gut microbiota to produce short chain fatty acids, including butyrate. Butyrate is efficiently absorbed by colonocytes and is utilized as energy source. Butyrate-mediated activation of the peroxisome proliferator-activated receptor-gamma (PPAR-γ) induces β-oxidation and consumption of oxygen, thus facilitating the establishment of anaerobic conditions that are required for the growth and function of several anaerobic gut commensals (57). In the specialized enteroendocrine cell (EEC), butyrate binds free fatty acid receptors (FFAR) FFAR2 and FFAR3 and regulates gut hormone release (73). Butyrate also acts as histone deacetylase (HDAC) inhibitor to regulate gene expression in EEC (76) and enterocytes (63). After absorption and utilization by colonocytes, the residual butyrate is first drained into the portal circulation, and then into the peripheral systemic circulation (29). In the systemic circulation, butyrate may regulate thermogenesis in brown adipose tissue (66) and β-cell function in pancreas (82).

Therefore, SCFAs (including butyrate) may play important roles in metabolic control, particularly *via* regulation of EECs and adipocyte lipolysis. However, specific targeting of the GPCRs in humans might be difficult due to their complex chemistry and diverse functions in different tissues (84). Additionally, applications might be limited by the lack of concordance between mouse and human studies that can possibly be explained by differences in dose, route and duration of administration, discrepancy between experimental models for diabetes and human diabetes, and specific intestinal environments in the different hosts. Nevertheless, the animal studies suggest that butyrate influences the regulation of glucose metabolism through multiple pathways that, if further characterized and validated in humans, can possibly be harnessed for the development of therapeutic strategies (**Figure 2**).

## Restoration of Butyrate in Type 2 Diabetes

Restoration of the intestinal levels of butyrate might be a novel strategy for the treatment of T2D, that could also be added to conventional therapy with lifestyle management and glucose-lowering drugs. In recent years, a number of studies have attempted to replenish butyrate levels and butyrate-producers in the gut using different approaches as discussed below: direct supplementation of butyrate or butyrate-producing bacteria, dietary supplementation of fibers to feed microbial butyrate production, and fecal microbiota transplantation.

### Supplementation of Butyrate

Butyrate can be supplemented as sodium conjugate or as tributyrin (a triglyceride in which glycerol is esterified with three butyrate molecules). In mice with diet-induced obesity, oral supplementation of 400mg/kg of butyrate improved glucose tolerance and increased the expression of phosphorylated adenosine monophosphate kinase (AMPK) as well as glucose transporter-4 in the adipose tissue, and reversed some of the gut microbiota alterations caused by the high-fat diet (HFD) (85). In another mouse study, addition of 5% butyrate to HFD increased energy expenditure, improved insulin sensitivity, and induced adaptive thermogenesis in BAT followed by increased AMPK activity and mitochondrial biogenesis in muscle cells (66). As an alternative, tributyrin supplementation in diet-induced obese mice was also shown to improve glucose tolerance and inflammatory status (86), indicating that direct supplementation of butyrate might have beneficial effects on both metabolic and inflammatory parameters relevant for the pathophysiology of T2D.

With convincing results in mouse studies, butyrate supplementation was also tested in individuals with and without metabolic syndrome who were given 4g sodium butyrate in capsules for a period of 4 weeks (80). In this study, butyrate supplementation did not increase butyrate levels either in feces or plasma, but it improved both peripheral and hepatic insulin sensitivity in individuals without metabolic syndrome. In another study, oral butyrate supplementation at the same dose improved the inflammatory status in individuals with metabolic syndrome, but no effect on insulin sensitivity was measured in this study (87). Additional studies might be required to determine effective doses of butyrate in humans, or other methods of administration and delivery of butyrate to the colonic epithelium that mimics the production by the gut microbiota.

### Butyrate-Producing Bacteria

Live bacteria that provide health benefits when consumed are generally called probiotics, and traditional *Lactobacillus* probiotics have demonstrated some efficacy for hyperglycemia and insulin sensitivity in human cohorts (88–91). In two independent studies, supplementation of *Lactobacillus paracasei* or *Bifidobacterium bifidum* to healthy individuals increased fecal butyrate levels (92, 93), indicating that traditional probiotics may modulate the activity of butyrate producers. However, the intestinal microbes that have been found as decreased in T2D in metagenomics studies are not traditional probiotics, and are being explored to produce next-generation probiotics (NGPs) (94, 95). For the butyrate producers, oral administration of *Clostridium butyricum* to mice lacking the leptin receptor, or to mice on HFD injected with streptozotocin to induce diabetes, was shown to improve oral glucose tolerance and insulin levels, and to increase the abundance of butyrate producers and fecal butyrate levels (96). In another study, oral administration of *Eubacterium hallii* to mice lacking the leptin receptor improved insulin sensitivity and increased energy expenditure (97). However, administration of *E. hallii strain* L2-7 (now reclassified as *Anaerobutyricum soehngenii*) to individuals with insulin resistance improved insulin sensitivity only in individuals with a specific gut microbiota at baseline (98), reflecting both the resilience of the human gut microbiota and the ecological interactions of commensal microbes in the communities that might be species-specific. To produce effective NGPs, advanced data-driven metagenomics approaches (99) and specific isolation efforts might be required to develop synthetic microbial communities targeted to produce butyrate.

### Dietary Fiber

Since butyrate-producing bacteria feed upon dietary fiber, dietary supplementation with fiber may provide a feasible option to increase the levels and the activity of the bacteria, and increase the intestinal levels of butyrate. In a randomized clinical study by Zhao et al., supplementation of a mix of dietary fibers to individuals with T2D improved glycemic parameters, accompanied by increased abundance of acetate- and butyrate-producing bacteria and increased fecal levels of acetate and butyrate (100). In another study, combining a mix of butyrate-producing species (*E. hallii*, *Clostridium beijerinckii* and *C. butyricum*), with other gut bacteria (*A. muciniphila* and *Bifidobacterium infantis*) and inulin as fermentable fiber modestly increased butyrate levels and improved oral glucose tolerance and glycated hemoglobin levels in individuals with T2D (101). Finally, dietary supplementation of inulin along with sodium butyrate in capsules for 45 days improved fasting glucose and waist-to-hip ratio in individuals with T2D (75). These studies clearly indicate that dietary fiber itself or in combination with NGPs or butyrate can improve glucose control in T2D. However, strategies to maintain patient compliance and investigations of long-term effects of these supplements are still warranted. Additionally, as it is now evident that the baseline gut microbiota is a strong predictor of success for dietary interventions (102, 103), probiotic administrations (89) and microbiota transplantations (104), stratification of individuals with T2D based on their microbiota may help to achieve better metabolic outcomes.

### Microbiota Transplantation

Microbiota transplants from mouse models (105) and humans (45, 106) into GF mice have successfully demonstrated the transmissibility of donor’s phenotypes. Therefore, human-to-human fecal microbiota transplants (FMT) [that have shown unprecedented success for the treatment of *Clostridium difficile* infections (107)] have recently been attempted for the treatment of T2D. When insulin resistant individuals were administered with duodenal infusion their own fecal microbiota (autologous) or fecal microbiota from a healthy lean donor (allogenic), the allogenic group displayed an improved insulin sensitivity (104, 108). The improved phenotype was observed 6 weeks after transplant in both studies, but not 18 weeks post-FMT (104). Change in butyrate producers (such as *Roseburia*, *Eubacterium* and *Butyrivibiro*) in feces and in the small intestine was observed in both studies, but increase in butyrate levels were observed only in one (108). In another study, daily cellulose supplementation after a single-dose oral FMT in individuals with metabolic syndrome improved insulin sensitivity 6 weeks after treatment compared to baseline. The authors found that this outcome was linked to higher GLP-1 secretion and better engraftment of the donor microbiota; however, they did not report significant changes for either the proportions of butyrate producers or fecal butyrate levels (109). Overall, the studies on FMT indicate that, while this procedure may improve insulin sensitivity in the short-term, the host gut microbiota is resilient enough to drift away the foreign microbial community in the long-term. In addition, while FMT is generally associated with mild side-effects, major adverse events have also been reported (110). These observations question the feasibility and applicability of FMT as a way of treatment for T2D.

## Butyrate as Therapy – Conclusions

Butyrate has long been known as a microbial fermentation product of dietary fibers in the gut, and references of butyrate-producing bacteria isolated from dietary sources emerged already in late 1940 (111). The recent association of T2D with reduction of butyrate-producing bacteria has spurred interest to explore the therapeutic potential of butyrate for the treatment of T2D but, while the results of experimental studies overall look promising, human interventions have only shown positive outcomes in the short term, and might have important limitations. In particular, current studies based on the metagenomic profiling of DNA are not able to determine the activity of butyrate producers in the human gut. Probiotics, NGPs and fiber supplementations might be successful strategies to increase butyrate-producing bacteria and improve hyperglycemia and insulin resistance, but their effects might be dependent on the individualized gut microbiota at baseline (responders *vs*. non-responders) and/or mediated by multiple undefined mechanisms besides butyrate production. FMT seems promising for the restoration of the gut microbiota and to improve insulin sensitivity, but it is impractical to perform such a highly invasive procedure in humans for short-term benefits. Future studies are required to gain a better understanding of the intestinal conditions that might influence butyrate production in individuals with T2D, in relation both to the diet and the individualized gut microbiota; for example, ingestible electronic capsules able to monitor microbial fermentations directly in the gut (112) could be used to characterize intestinal conditions, responses to fibers and microbiota profiles linked to homeostatic butyrate production. Furthermore, robust methods for the measurement of butyrate, tracer studies and live-detection of butyrate-producing bacteria [for example, by flow cytometry (113)] might help to strengthen the association of butyrate with T2D and identify new potential NGPs or synthetic microbial communities for butyrate-based management of T2D.

## Author Contributions

TA conceptualized the manuscript. TA and VT wrote the manuscript. All authors contributed to the article and approved the submitted version.

## Funding

This work was supported by Novo Nordisk Foundation (Grant no. NNF15OC0016798).

## Conflict of Interest

The authors declare that the research was conducted in the absence of any commercial or financial relationships that could be construed as a potential conflict of interest.

## Publisher’s Note

All claims expressed in this article are solely those of the authors and do not necessarily represent those of their affiliated organizations, or those of the publisher, the editors and the reviewers. Any product that may be evaluated in this article, or claim that may be made by its manufacturer, is not guaranteed or endorsed by the publisher.

## References

[1] Cho NH Shaw JE Karuranga S Huang Y da Rocha Fernandes JD Ohlrogge AW. IDF Diabetes Atlas: Global Estimates of Diabetes Prevalence for 2017 and Projections for 2045. Diabetes Res Clin Pract (2018) 138:271–81. doi: 10.1016/j.diabres.2018.02.023 29496507

[2] Engler C Leo M Pfeifer B Juchum M Chen-Koenig D Poelzl K. Long-Term Trends in the Prescription of Antidiabetic Drugs: Real-World Evidence From the Diabetes Registry Tyrol 2012-2018. BMJ Open Diabetes Res Care (2020) 8:e001279. doi: 10.1136/bmjdrc-2020-001279 PMC7467522 32873600

[3] O'Hara AM Shanahan F. The Gut Flora as a Forgotten Organ. EMBO Rep (2006) 7:688–93. doi: 10.1038/sj.embor.7400731 PMC1500832 16819463

[4] Gill SR Pop M Deboy RT Eckburg PB Turnbaugh PJ Samuel BS. Metagenomic Analysis of the Human Distal Gut Microbiome. Science (2006) 312:1355–9. doi: 10.1126/science.1124234 PMC3027896 16741115

[5] Eckburg PB Bik EM Bernstein CN Purdom E Dethlefsen L Sargent M. Diversity of the Human Intestinal Microbial Flora. Science (2005) 308:1635–8. doi: 10.1126/science.1110591 PMC1395357 15831718

[6] Suau A Bonnet R Sutren M Godon JJ Gibson GR Collins MD. Direct Analysis of Genes Encoding 16S rRNA From Complex Communities Reveals Many Novel Molecular Species Within the Human Gut. Appl Environ Microbiol (1999) 65:4799–807. doi: 10.1128/AEM.65.11.4799-4807.1999 PMC91647 10543789

[7] Goodman AL Kallstrom G Faith JJ Reyes A Moore A Dantas G. Extensive Personal Human Gut Microbiota Culture Collections Characterized and Manipulated in Gnotobiotic Mice. Proc Natl Acad Sci USA (2011) 108:6252–7. doi: 10.1073/pnas.1102938108 PMC3076821 21436049

[8] Salonen A Salojarvi J Lahti L de Vos WM. The Adult Intestinal Core Microbiota Is Determined by Analysis Depth and Health Status. Clin Microbiol Infect (2012) 18(Suppl 4):16–20. doi: 10.1111/j.1469-0691.2012.03855.x 22647042

[9] Turnbaugh PJ Hamady M Yatsunenko T Cantarel BL Duncan A Ley RE. A Core Gut Microbiome in Obese and Lean Twins. Nature (2009) 457:480–4. doi: 10.1038/nature07540 PMC2677729 19043404

[10] Qin J Li R Raes J Arumugam M Burgdorf KS Manichanh C. A Human Gut Microbial Gene Catalogue Established by Metagenomic Sequencing. Nature (2010) 464:59–65. doi: 10.1038/nature08821 20203603 PMC3779803

[11] Human Microbiome Project C. Structure, Function and Diversity of the Healthy Human Microbiome. Nature (2012) 486:207–14. doi: 10.1038/nature11234 PMC3564958 22699609

[12] Falony G Joossens M Vieira-Silva S Wang J Darzi Y Faust K. Population-Level Analysis of Gut Microbiome Variation. Science (2016) 352:560–4. doi: 10.1126/science.aad3503 27126039

[13] Li J Jia H Cai X Zhong H Feng Q Sunagawa S. An Integrated Catalog of Reference Genes in the Human Gut Microbiome. Nat Biotechnol (2014) 32:834–41. doi: 10.1038/nbt.2942 24997786

[14] Yatsunenko T Rey FE Manary MJ Trehan I Dominguez-Bello MG Contreras M. Human Gut Microbiome Viewed Across Age and Geography. Nature (2012) 486:222–7. doi: 10.1038/nature11053 PMC3376388 22699611

[15] Rampelli S Schnorr SL Consolandi C Turroni S Severgnini M Peano C. Metagenome Sequencing of the Hadza Hunter-Gatherer Gut Microbiota. Curr Biol (2015) 25:1682–93. doi: 10.1016/j.cub.2015.04.055 25981789

[16] Deschasaux M Bouter KE Prodan A Levin E Groen AK Herrema H. Depicting the Composition of Gut Microbiota in a Population With Varied Ethnic Origins But Shared Geography. Nat Med (2018) 24:1526–31. doi: 10.1038/s41591-018-0160-1 30150717

[17] Brooks AW Priya S Blekhman R Bordenstein SR. Gut Microbiota Diversity Across Ethnicities in the United States. PLoS Biol (2018) 16:e2006842. doi: 10.1371/journal.pbio.2006842 30513082 PMC6279019

[18] Duvallet C Gibbons SM Gurry T Irizarry RA Alm EJ. Meta-Analysis of Gut Microbiome Studies Identifies Disease-Specific and Shared Responses. Nat Commun (2017) 8:1784. doi: 10.1038/s41467-017-01973-8 29209090 PMC5716994

[19] Karlsson FH Tremaroli V Nookaew I Bergstrom G Behre CJ Fagerberg B. Gut Metagenome in European Women With Normal, Impaired and Diabetic Glucose Control. Nature (2013) 498:99–103. doi: 10.1038/nature12198 23719380

[20] Qin J Li Y Cai Z Li S Zhu J Zhang F. A Metagenome-Wide Association Study of Gut Microbiota in Type 2 Diabetes. Nature (2012) 490:55–60. doi: 10.1038/nature11450 23023125

[21] Bar N Korem T Weissbrod O Zeevi D Rothschild D Leviatan S. A Reference Map of Potential Determinants for the Human Serum Metabolome. Nature (2020) 588:135–40. doi: 10.1038/s41586-020-2896-2 33177712

[22] Krautkramer KA Fan J Backhed F. Gut Microbial Metabolites as Multi-Kingdom Intermediates. Nat Rev Microbiol (2021) 19:77–94. doi: 10.1038/s41579-020-0438-4 32968241

[23] Koeth RA Wang Z Levison BS Buffa JA Org E Sheehy BT. Intestinal Microbiota Metabolism of L-Carnitine, A Nutrient in Red Meat, Promotes Atherosclerosis. Nat Med (2013) 19:576–85. doi: 10.1038/nm.3145 PMC3650111 23563705

[24] Koh A Molinaro A Stahlman M Khan MT Schmidt C Manneras-Holm L. Microbially Produced Imidazole Propionate Impairs Insulin Signaling Through Mtorc1. Cell (2018) 175:947–61.e17. doi: 10.1016/j.cell.2018.09.055 30401435

[25] de Mello VD Paananen J Lindstrom J Lankinen MA Shi L Kuusisto J. Indolepropionic Acid and Novel Lipid Metabolites Are Associated With a Lower Risk of Type 2 Diabetes in the Finnish Diabetes Prevention Study. Sci Rep (2017) 7:46337. doi: 10.1038/srep46337 28397877 PMC5387722

[26] Fleming SE Rodriguez MA. Influence of Dietary Fiber on Fecal Excretion of Volatile Fatty Acids by Human Adults. J Nutr (1983) 113:1613–25. doi: 10.1093/jn/113.8.1613 6308193

[27] Flint HJ Scott KP Louis P Duncan SH. The Role of the Gut Microbiota in Nutrition and Health. Nat Rev Gastroenterol Hepatol (2012) 9:577–89. doi: 10.1038/nrgastro.2012.156 22945443

[28] Zhang L Liu C Jiang Q Yin Y. Butyrate in Energy Metabolism: There Is Still More to Learn. Trends Endocrinol Metab (2021) 32:159–69. doi: 10.1016/j.tem.2020.12.003 33461886

[29] Cummings JH Pomare EW Branch WJ Naylor CP Macfarlane GT. Short Chain Fatty Acids in Human Large Intestine, Portal, Hepatic and Venous Blood. Gut (1987) 28:1221–7. doi: 10.1136/gut.28.10.1221 PMC1433442 3678950

[30] Zaneveld JR McMinds R Vega Thurber R. Stress and Stability: Applying the Anna Karenina Principle to Animal Microbiomes. Nat Microbiol (2017) 2:17121. doi: 10.1038/nmicrobiol.2017.121 28836573

[31] Bhute SS Suryavanshi MV Joshi SM Yajnik CS Shouche YS Ghaskadbi SS. Gut Microbial Diversity Assessment of Indian Type-2-Diabetics Reveals Alterations in Eubacteria, Archaea, and Eukaryotes. Front Microbiol (2017) 8:214. doi: 10.3389/fmicb.2017.00214 28261173 PMC5306211

[32] Doumatey AP Adeyemo A Zhou J Lei L Adebamowo SN Adebamowo C. Gut Microbiome Profiles Are Associated With Type 2 Diabetes in Urban Africans. Front Cell Infect Microbiol (2020) 10:63. doi: 10.3389/fcimb.2020.00063 32158702 PMC7052266

[33] Anhe FF Jensen BAH Varin TV Servant F Van Blerk S Richard D. Type 2 Diabetes Influences Bacterial Tissue Compartmentalisation in Human Obesity. Nat Metab (2020) 2:233–42. doi: 10.1038/s42255-020-0178-9 32694777

[34] Sanna S van Zuydam NR Mahajan A Kurilshikov A Vich Vila A Vosa U. Causal Relationships Among the Gut Microbiome, Short-Chain Fatty Acids and Metabolic Diseases. Nat Genet (2019) 51:600–5. doi: 10.1038/s41588-019-0350-x PMC6441384 30778224

[35] Reitmeier S Kiessling S Clavel T List M Almeida EL Ghosh TS. Arrhythmic Gut Microbiome Signatures Predict Risk of Type 2 Diabetes. Cell Host Microbe (2020) 28:258–72.e6. doi: 10.1016/j.chom.2020.06.004 32619440

[36] Gurung M Li Z You H Rodrigues R Jump DB Morgun A. Role of Gut Microbiota in Type 2 Diabetes Pathophysiology. EBioMedicine (2020) 51:102590. doi: 10.1016/j.ebiom.2019.11.051 31901868 PMC6948163

[37] Cunningham AL Stephens JW Harris DA. Gut Microbiota Influence in Type 2 Diabetes Mellitus (T2DM). Gut Pathog (2021) 13:50. doi: 10.1186/s13099-021-00446-0 34362432 PMC8343927

[38] Maier L Pruteanu M Kuhn M Zeller G Telzerow A Anderson EE. Extensive Impact of Non-Antibiotic Drugs on Human Gut Bacteria. Nature (2018) 555:623–8. doi: 10.1038/nature25979 PMC6108420 29555994

[39] Lee H Lee Y Kim J An J Lee S Kong H. Modulation of the Gut Microbiota by Metformin Improves Metabolic Profiles in Aged Obese Mice. Gut Microbes (2018) 9:155–65. doi: 10.1080/19490976.2017.1405209 PMC5989809 29157127

[40] Shin NR Lee JC Lee HY Kim MS Whon TW Lee MS. An Increase in the Akkermansia Spp. Population Induced by Metformin Treatment Improves Glucose Homeostasis in Diet-Induced Obese Mice. Gut (2014) 63:727–35. doi: 10.1136/gutjnl-2012-303839 23804561

[41] Cani PD de Vos WM. Next-Generation Beneficial Microbes: The Case of Akkermansia Muciniphila. Front Microbiol (2017) 8:1765. doi: 10.3389/fmicb.2017.01765 29018410 PMC5614963

[42] Forslund K Hildebrand F Nielsen T Falony G Le Chatelier E Sunagawa S. Disentangling Type 2 Diabetes and Metformin Treatment Signatures in the Human Gut Microbiota. Nature (2015) 528:262–6. doi: 10.1038/nature15766 PMC4681099 26633628

[43] Wu H Tremaroli V Schmidt C Lundqvist A Olsson LM Kramer M. The Gut Microbiota in Prediabetes and Diabetes: A Population-Based Cross-Sectional Study. Cell Metab (2020) 32:379–90.e3. doi: 10.1016/j.cmet.2020.06.011 32652044

[44] de la Cuesta-Zuluaga J Mueller NT Corrales-Agudelo V Velasquez-Mejia EP Carmona JA Abad JM. Metformin Is Associated With Higher Relative Abundance of Mucin-Degrading Akkermansia Muciniphila and Several Short-Chain Fatty Acid-Producing Microbiota in the Gut. Diabetes Care (2017) 40:54–62. doi: 10.2337/dc16-1324 27999002

[45] Wu H Esteve E Tremaroli V Khan MT Caesar R Manneras-Holm L. Metformin Alters the Gut Microbiome of Individuals With Treatment-Naive Type 2 Diabetes, Contributing to the Therapeutic Effects of the Drug. Nat Med (2017) 23:850–8. doi: 10.1038/nm.4345 28530702

[46] Pryor R Norvaisas P Marinos G Best L Thingholm LB Quintaneiro LM. Host-Microbe-Drug-Nutrient Screen Identifies Bacterial Effectors of Metformin Therapy. Cell (2019) 178:1299–312.e29. doi: 10.1016/j.cell.2019.08.003 31474368 PMC6736778

[47] Thingholm LB Ruhlemann MC Koch M Fuqua B Laucke G Boehm R. Obese Individuals With and Without Type 2 Diabetes Show Different Gut Microbial Functional Capacity and Composition. Cell Host Microbe (2019) 26:252–64.e10. doi: 10.1016/j.chom.2019.07.004 31399369 PMC7720933

[48] Tabak AG Herder C Rathmann W Brunner EJ Kivimaki M. Prediabetes: A High-Risk State for Diabetes Development. Lancet (2012) 379:2279–90. doi: 10.1016/S0140-6736(12)60283-9 PMC3891203 22683128

[49] Allin KH Tremaroli V Caesar R Jensen BAH Damgaard MTF Bahl MI. Aberrant Intestinal Microbiota in Individuals With Prediabetes. Diabetologia (2018) 61:810–20. doi: 10.1007/s00125-018-4550-1 PMC6448993 29379988

[50] Adachi K Sugiyama T Yamaguchi Y Tamura Y Izawa S Hijikata Y. Gut Microbiota Disorders Cause Type 2 Diabetes Mellitus and Homeostatic Disturbances in Gut-Related Metabolism in Japanese Subjects. J Clin Biochem Nutr (2019) 64:231–8. doi: 10.3164/jcbn.18-101 PMC6529700 31138957

[51] Zhao L Lou H Peng Y Chen S Zhang Y Li X. Comprehensive Relationships Between Gut Microbiome and Faecal Metabolome in Individuals With Type 2 Diabetes and Its Complications. Endocrine (2019) 66:526–37. doi: 10.1007/s12020-019-02103-8 31591683

[52] Louis P Flint HJ. Formation of Propionate and Butyrate by the Human Colonic Microbiota. Environ Microbiol (2017) 19:29–41. doi: 10.1111/1462-2920.13589 27928878

[53] Pryde SE Duncan SH Hold GL Stewart CS Flint HJ. The Microbiology of Butyrate Formation in the Human Colon. FEMS Microbiol Lett (2002) 217:133–9. doi: 10.1111/j.1574-6968.2002.tb11467.x 12480096

[54] Vital M Karch A Pieper DH. Colonic Butyrate-Producing Communities in Humans: An Overview Using Omics Data. mSystems (2017) 2:e00130–17. doi: 10.1128/mSystems.00130-17 PMC5715108 29238752

[55] Louis P Young P Holtrop G Flint HJ. Diversity of Human Colonic Butyrate-Producing Bacteria Revealed by Analysis of the Butyryl-CoA:acetate CoA-Transferase Gene. Environ Microbiol (2010) 12:304–14. doi: 10.1111/j.1462-2920.2009.02066.x 19807780

[56] Vital M Howe AC Tiedje JM. Revealing the Bacterial Butyrate Synthesis Pathways by Analyzing (Meta)Genomic Data. mBio (2014) 5:e00889. doi: 10.1128/mBio.00889-14 24757212 PMC3994512

[57] Byndloss MX Olsan EE Rivera-Chavez F Tiffany CR Cevallos SA Lokken KL. Microbiota-Activated PPAR-Gamma Signaling Inhibits Dysbiotic Enterobacteriaceae Expansion. Science (2017) 357:570–5. doi: 10.1126/science.aam9949 PMC5642957 28798125

[58] Boets E Gomand SV Deroover L Preston T Vermeulen K De Preter V. Systemic Availability and Metabolism of Colonic-Derived Short-Chain Fatty Acids in Healthy Subjects: A Stable Isotope Study. J Physiol (2017) 595:541–55. doi: 10.1113/JP272613 PMC5233652 27510655

[59] Donohoe DR Garge N Zhang X Sun W O'Connell TM Bunger MK. The Microbiome and Butyrate Regulate Energy Metabolism and Autophagy in the Mammalian Colon. Cell Metab (2011) 13:517–26. doi: 10.1016/j.cmet.2011.02.018 PMC3099420 21531334

[60] Wichmann A Allahyar A Greiner TU Plovier H Lunden GO Larsson T. Microbial Modulation of Energy Availability in the Colon Regulates Intestinal Transit. Cell Host Microbe (2013) 14:582–90. doi: 10.1016/j.chom.2013.09.012 24237703

[61] Yuille S Reichardt N Panda S Dunbar H Mulder IE. Human Gut Bacteria as Potent Class I Histone Deacetylase Inhibitors *In Vitro* Through Production of Butyric Acid and Valeric Acid. PLoS One (2018) 13:e0201073. doi: 10.1371/journal.pone.0201073 30052654 PMC6063406

[62] Ling C Ronn T. Epigenetics in Human Obesity and Type 2 Diabetes. Cell Metab (2019) 29:1028–44. doi: 10.1016/j.cmet.2019.03.009 PMC6509280 30982733

[63] Noureldein MH Bitar S Youssef N Azar S Eid AA. Butyrate Modulates Diabetes-Linked Gut Dysbiosis: Epigenetic and Mechanistic Modifications. J Mol Endocrinol (2020) 64:29–42. doi: 10.1530/JME-19-0132 31770101

[64] Chriett S Zerzaihi O Vidal H Pirola L. The Histone Deacetylase Inhibitor Sodium Butyrate Improves Insulin Signalling in Palmitate-Induced Insulin Resistance in L6 Rat Muscle Cells Through Epigenetically-Mediated Up-Regulation of Irs1. Mol Cell Endocrinol (2017) 439:224–32. doi: 10.1016/j.mce.2016.09.006 27619406

[65] Henagan TM Stefanska B Fang Z Navard AM Ye J Lenard NR. Sodium Butyrate Epigenetically Modulates High-Fat Diet-Induced Skeletal Muscle Mitochondrial Adaptation, Obesity and Insulin Resistance Through Nucleosome Positioning. Br J Pharmacol (2015) 172:2782–98. doi: 10.1111/bph.13058 PMC4439875 25559882

[66] Gao Z Yin J Zhang J Ward RE Martin RJ Lefevre M. Butyrate Improves Insulin Sensitivity and Increases Energy Expenditure in Mice. Diabetes (2009) 58:1509–17. doi: 10.2337/db08-1637 PMC2699871 19366864

[67] Khan S Jena G. Sodium Butyrate Reduces Insulin-Resistance, Fat Accumulation and Dyslipidemia in Type-2 Diabetic Rat: A Comparative Study With Metformin. Chem Biol Interact (2016) 254:124–34. doi: 10.1016/j.cbi.2016.06.007 27270450

[68] Brown AJ Goldsworthy SM Barnes AA Eilert MM Tcheang L Daniels D. The Orphan G Protein-Coupled Receptors GPR41 and GPR43 Are Activated by Propionate and Other Short Chain Carboxylic Acids. J Biol Chem (2003) 278:11312–9. doi: 10.1074/jbc.M211609200 12496283

[69] Tunaru S Kero J Schaub A Wufka C Blaukat A Pfeffer K. PUMA-G and HM74 Are Receptors for Nicotinic Acid and Mediate Its Anti-Lipolytic Effect. Nat Med (2003) 9:352–5. doi: 10.1038/nm824 12563315

[70] Husted AS Trauelsen M Rudenko O Hjorth SA Schwartz TW. GPCR-Mediated Signaling of Metabolites. Cell Metab (2017) 25:777–96. doi: 10.1016/j.cmet.2017.03.008 28380372

[71] Petersen N Reimann F Bartfeld S Farin HF Ringnalda FC Vries RG. Generation of L Cells in Mouse and Human Small Intestine Organoids. Diabetes (2014) 63:410–20. doi: 10.2337/db13-0991 PMC4306716 24130334

[72] Arora T Vanslette AM Hjorth SA Backhed F. Microbial Regulation of Enteroendocrine Cells. Med-Cambridge (2021) 2:553–70. doi: 10.1016/j.medj.2021.03.018 35590233

[73] Tolhurst G Heffron H Lam YS Parker HE Habib AM Diakogiannaki E. Short-Chain Fatty Acids Stimulate Glucagon-Like Peptide-1 Secretion *via* the G-Protein-Coupled Receptor FFAR2. Diabetes (2012) 61:364–71. doi: 10.2337/db11-1019 PMC3266401 22190648

[74] Aroda VR Bain SC Cariou B Piletic M Rose L Axelsen M. Efficacy and Safety of Once-Weekly Semaglutide *Versus* Once-Daily Insulin Glargine as Add-on to Metformin (With or Without Sulfonylureas) in Insulin-Naive Patients With Type 2 Diabetes (SUSTAIN 4): A Randomised, Open-Label, Parallel-Group, Multicentre, Multinational, Phase 3a Trial. Lancet Diabetes Endo (2017) 5:355–66. doi: 10.1016/S2213-8587(17)30085-2 28344112

[75] Roshanravan N Mahdavi R Alizadeh E Jafarabadi MA Hedayati M Ghavami A. Effect of Butyrate and Inulin Supplementation on Glycemic Status, Lipid Profile and Glucagon-Like Peptide 1 Level in Patients With Type 2 Diabetes: A Randomized Double-Blind, Placebo-Controlled Trial. Horm Metab Res (2017) 49:886–91. doi: 10.1055/s-0043-119089 28962046

[76] Larraufie P Martin-Gallausiaux C Lapaque N Dore J Gribble FM Reimann F. SCFAs Strongly Stimulate PYY Production in Human Enteroendocrine Cells. Sci Rep (2018) 8:74. doi: 10.1038/s41598-017-18259-0 29311617 PMC5758799

[77] Dobbins R Byerly R Gaddy R Gao F Mahar K Napolitano A. GSK256073 Acutely Regulates NEFA Levels *via* HCA2 Agonism But Does Not Achieve Durable Glycaemic Control in Type 2 Diabetes. A Randomised Trial. Eur J Pharmacol (2015) 755:95–101. doi: 10.1016/j.ejphar.2015.03.005 25773496

[78] Dobbins RL Shearn SP Byerly RL Gao FF Mahar KM Napolitano A. GSK256073, a Selective Agonist of G-Protein Coupled Receptor 109A (GPR109A) Reduces Serum Glucose in Subjects With Type 2 Diabetes Mellitus. Diabetes Obes Metab (2013) 15:1013–21. doi: 10.1111/dom.12132 23701262

[79] Kimura I Ozawa K Inoue D Imamura T Kimura K Maeda T. The Gut Microbiota Suppresses Insulin-Mediated Fat Accumulation via the Short-Chain Fatty Acid Receptor GPR43. Nat Commun (2013) 4:1829. doi: 10.1038/ncomms2852 23652017 PMC3674247

[80] Bouter K Bakker GJ Levin E Hartstra AV Kootte RS Udayappan SD. Differential Metabolic Effects of Oral Butyrate Treatment in Lean *Versus* Metabolic Syndrome Subjects. Clin Transl Gastroenterol (2018) 9:155. doi: 10.1038/s41424-018-0025-4 29799027 PMC5968024

[81] Hu S Kuwabara R de Haan BJ Smink AM de Vos P. Acetate and Butyrate Improve Beta-Cell Metabolism and Mitochondrial Respiration Under Oxidative Stress. Int J Mol Sci (2020) 21:1542. doi: 10.3390/ijms21041542 PMC7073211 32102422

[82] Matheus VA Monteiro L Oliveira RB Maschio DA Collares-Buzato CB. Butyrate Reduces High-Fat Diet-Induced Metabolic Alterations, Hepatic Steatosis and Pancreatic Beta Cell and Intestinal Barrier Dysfunctions in Prediabetic Mice. Exp Biol Med (Maywood) (2017) 242:1214–26. doi: 10.1177/1535370217708188 PMC5476343 28504618

[83] de Groot PF Nikolic T Imangaliyev S Bekkering S Duinkerken G Keij FM. Oral Butyrate Does Not Affect Innate Immunity and Islet Autoimmunity in Individuals With Longstanding Type 1 Diabetes: A Randomised Controlled Trial. Diabetologia (2020) 63:597–610. doi: 10.1007/s00125-019-05073-8 31915895

[84] Milligan G Shimpukade B Ulven T Hudson BD. Complex Pharmacology of Free Fatty Acid Receptors. Chem Rev (2017) 117:67–110. doi: 10.1021/acs.chemrev.6b00056 27299848

[85] Gao F Lv YW Long J Chen JM He JM Ruan XZ. Butyrate Improves the Metabolic Disorder and Gut Microbiome Dysbiosis in Mice Induced by a High-Fat Diet. Front Pharmacol (2019) 10:1040. doi: 10.3389/fphar.2019.01040 31607907 PMC6761375

[86] Sato FT Yap YA Crisma AR Portovedo M Murata GM Hirabara SM. Tributyrin Attenuates Metabolic and Inflammatory Changes Associated With Obesity Through a GPR109A-Dependent Mechanism. Cells (2020) 9. doi: 10.3390/cells9092007 PMC7563536 32882837

[87] Cleophas MCP Ratter JM Bekkering S Quintin J Schraa K Stroes ES. Effects of Oral Butyrate Supplementation on Inflammatory Potential of Circulating Peripheral Blood Mononuclear Cells in Healthy and Obese Males. Sci Rep (2019) 9:775. doi: 10.1038/s41598-018-37246-7 30692581 PMC6349871

[88] Andreasen AS Larsen N Pedersen-Skovsgaard T Berg RM Moller K Svendsen KD. Effects of Lactobacillus Acidophilus NCFM on Insulin Sensitivity and the Systemic Inflammatory Response in Human Subjects. Br J Nutr (2010) 104:1831–8. doi: 10.1017/S0007114510002874 20815975

[89] Mobini R Tremaroli V Stahlman M Karlsson F Levin M Ljungberg M. Metabolic Effects of Lactobacillus Reuteri DSM 17938 in People With Type 2 Diabetes: A Randomized Controlled Trial. Diabetes Obes Metab (2017) 19:579–89. doi: 10.1111/dom.12861 28009106

[90] Simon MC Strassburger K Nowotny B Kolb H Nowotny P Burkart V. Intake of Lactobacillus Reuteri Improves Incretin and Insulin Secretion in Glucose-Tolerant Humans: A Proof of Concept. Diabetes Care (2015) 38:1827–34. doi: 10.2337/dc14-2690 26084343

[91] Sun J Buys NJ. Glucose- and Glycaemic Factor-Lowering Effects of Probiotics on Diabetes: A Meta-Analysis of Randomised Placebo-Controlled Trials. Br J Nutr (2016) 115:1167–77. doi: 10.1017/S0007114516000076 26899960

[92] Ferrario C Taverniti V Milani C Fiore W Laureati M De Noni I. Modulation of Fecal Clostridiales Bacteria and Butyrate by Probiotic Intervention With Lactobacillus Paracasei DG Varies Among Healthy Adults. J Nutr (2014) 144:1787–96. doi: 10.3945/jn.114.197723 25332478

[93] Gargari G Taverniti V Balzaretti S Ferrario C Gardana C Simonetti P. Consumption of a Bifidobacterium Bifidum Strain for 4 Weeks Modulates Dominant Intestinal Bacterial Taxa and Fecal Butyrate in Healthy Adults. Appl Environ Microbiol (2016) 82:5850–9. doi: 10.1128/AEM.01753-16 PMC5038021 27451450

[94] Martin R Langella P. Emerging Health Concepts in the Probiotics Field: Streamlining the Definitions. Front Microbiol (2019) 10:1047. doi: 10.3389/fmicb.2019.01047 31164874 PMC6536656

[95] Langella P Guarner F Martin R. Editorial: Next-Generation Probiotics: From Commensal Bacteria to Novel Drugs and Food Supplements. Front Microbiol (2019) 10:1973. doi: 10.3389/fmicb.2019.01973 31507575 PMC6718915

[96] Jia L Li D Feng N Shamoon M Sun Z Ding L. Anti-Diabetic Effects of Clostridium Butyricum CGMCC0313.1 Through Promoting the Growth of Gut Butyrate-Producing Bacteria in Type 2 Diabetic Mice. Sci Rep (2017) 7:7046. doi: 10.1038/s41598-017-07335-0 28765642 PMC5539151

[97] Udayappan S Manneras-Holm L Chaplin-Scott A Belzer S Herrema S Dallinga-Thie S. Oral Treatment With Eubacterium Hallii Improves Insulin Sensitivity in Db/Db Mice. NPJ Biofilms Microbiomes (2016) 2:16009. doi: 10.1038/npjbiofilms.2016.9 28721246 PMC5515273

[98] Gilijamse PW Hartstra AV Levin E Wortelboer K Serlie MJ Ackermans MT. Treatment With Anaerobutyricum Soehngenii: A Pilot Study of Safety and Dose-Response Effects on Glucose Metabolism in Human Subjects With Metabolic Syndrome. NPJ Biofilms Microbiomes (2020) 6:16. doi: 10.1038/s41522-020-0127-0 32221294 PMC7101376

[99] Clark RL Connors BM Stevenson DM Hromada SE Hamilton JJ Amador-Noguez D. Design of Synthetic Human Gut Microbiome Assembly and Butyrate Production. Nat Commun (2021) 12:3254. doi: 10.1038/s41467-021-22938-y 34059668 PMC8166853

[100] Zhao L Zhang F Ding X Wu G Lam YY Wang X. Gut Bacteria Selectively Promoted by Dietary Fibers Alleviate Type 2 Diabetes. Science (2018) 359:1151–6. doi: 10.1126/science.aao5774 29590046

[101] Perraudeau F McMurdie P Bullard J Cheng A Cutcliffe C Deo A. Improvements to Postprandial Glucose Control in Subjects With Type 2 Diabetes: A Multicenter, Double Blind, Randomized Placebo-Controlled Trial of a Novel Probiotic Formulation. BMJ Open Diabetes Res Care (2020) 8:e001319. doi: 10.1136/bmjdrc-2020-001319 PMC7368581 32675291

[102] Jie Z Yu X Liu Y Sun L Chen P Ding Q. The Baseline Gut Microbiota Directs Dieting-Induced Weight Loss Trajectories. Gastroenterology (2021) 160:2029–42.e16. doi: 10.1053/j.gastro.2021.01.029 33482223

[103] Rodriguez J Hiel S Neyrinck AM Le Roy T Potgens SA Leyrolle Q. Discovery of the Gut Microbial Signature Driving the Efficacy of Prebiotic Intervention in Obese Patients. Gut (2020) 69:1975–87. doi: 10.1136/gutjnl-2019-319726 PMC7569399 32041744

[104] Kootte RS Levin E Salojarvi J Smits LP Hartstra AV Udayappan SD. Improvement of Insulin Sensitivity After Lean Donor Feces in Metabolic Syndrome Is Driven by Baseline Intestinal Microbiota Composition. Cell Metab (2017) 26:611–9.e6. 28978426 10.1016/j.cmet.2017.09.008

[105] Turnbaugh PJ Ley RE Mahowald MA Magrini V Mardis ER Gordon JI. An Obesity-Associated Gut Microbiome With Increased Capacity for Energy Harvest. Nature (2006) 444:1027–31. doi: 10.1038/nature05414 17183312

[106] Ridaura VK Faith JJ Rey FE Cheng J Duncan AE Kau AL. Gut Microbiota From Twins Discordant for Obesity Modulate Metabolism in Mice. Science (2013) 341:1241214. doi: 10.1126/science.1241214 24009397 PMC3829625

[107] van Nood E Vrieze A Nieuwdorp M Fuentes S Zoetendal EG de Vos WM. Duodenal Infusion of Donor Feces for Recurrent Clostridium Difficile. N Engl J Med (2013) 368:407–15. doi: 10.1056/NEJMoa1205037 23323867

[108] Vrieze A Van Nood E Holleman F Salojarvi J Kootte RS Bartelsman JF. Transfer of Intestinal Microbiota From Lean Donors Increases Insulin Sensitivity in Individuals With Metabolic Syndrome. Gastroenterology (2012) 143:913–6.e7. doi: 10.1053/j.gastro.2012.06.031 22728514

[109] Mocanu V Zhang Z Deehan EC Kao DH Hotte N Karmali S. Fecal Microbial Transplantation and Fiber Supplementation in Patients With Severe Obesity and Metabolic Syndrome: A Randomized Double-Blind, Placebo-Controlled Phase 2 Trial. Nat Med (2021) 27:1272–9. doi: 10.1038/s41591-021-01399-2 34226737

[110] Baxter M Colville A. Adverse Events in Faecal Microbiota Transplant: A Review of the Literature. J Hosp Infect (2016) 92:117–27. doi: 10.1016/j.jhin.2015.10.024 26803556

[111] Clark FM Dehr A. A Study of Butyric Acid-Producing Anaerobes Isolated From Spoiled Canned Tomatoes. Food Res (1947) 12:122–8. doi: 10.1111/j.1365-2621.1947.tb16401.x 20294337

[112] Kalantar-Zadeh K Berean KJ Burgell RE Muir JG Gibson PR. Intestinal Gases: Influence on Gut Disorders and the Role of Dietary Manipulations. Nat Rev Gastroenterol Hepatol (2019) 16:733–47. doi: 10.1038/s41575-019-0193-z 31520080

[113] Bruder LM Dorkes M Fuchs BM Ludwig W Liebl W. Flow Cytometric Sorting of Fecal Bacteria After *In Situ* Hybridization With Polynucleotide Probes. Syst Appl Microbiol (2016) 39:464–75. doi: 10.1016/j.syapm.2016.08.005 27665238

